# Designing multiepitope-based vaccine against *Eimeria* from immune mapped protein 1 (IMP-1) antigen using immunoinformatic approach

**DOI:** 10.1038/s41598-021-97880-6

**Published:** 2021-09-14

**Authors:** Thabile Madlala, Victoria T. Adeleke, Abiodun J. Fatoba, Moses Okpeku, Adebayo A. Adeniyi, Matthew A. Adeleke

**Affiliations:** 1 Discipline of Genetics, School of Life Sciences, University of KwaZulu-Natal, P/Bag X54001, Westville, Durban, 4000 South Africa; 2 Discipline of Chemical Engineering, University of KwaZulu-Natal, Howard Campus, Durban, 4041 South Africa; 3 Department of Chemistry, Faculty of Natural and Agricultural Sciences, University of the Free State, Bloemfontein, South Africa; 4 Department of Industrial Chemistry, Federal University Oye-Ekiti, Oye-Ekiti, Nigeria

**Keywords:** Computational biology and bioinformatics, Genetics, Immunology

## Abstract

Drug resistance against coccidiosis has posed a significant threat to chicken welfare and productivity worldwide, putting daunting pressure on the poultry industry to reduce the use of chemoprophylactic drugs and live vaccines in poultry to treat intestinal diseases. Chicken coccidiosis, caused by an apicomplexan parasite of *Eimeria* spp., is a significant challenge worldwide. Due to the experience of economic loss in production and prevention of the disease, development of cost-effective vaccines or drugs that can stimulate defence against multiple *Eimeria* species is imperative to control coccidiosis. This study explored *Eimeria* immune mapped protein-1 (IMP-1) to develop a multiepitope-based vaccine against coccidiosis by identifying antigenic T-cell and B-cell epitope candidates through immunoinformatic techniques. This resulted in the design of 7 CD8^+^, 21 CD4^+^ T-cell epitopes and 6 B-cell epitopes, connected using AAY, GPGPG and KK linkers to form a vaccine construct. A Cholera Toxin B (CTB) adjuvant was attached to the N-terminal of the multiepitope construct to improve the immunogenicity of the vaccine. The designed vaccine was assessed for immunogenicity (8.59968), allergenicity and physiochemical parameters, which revealed the construct molecular weight of 73.25 kDa, theoretical pI of 8.23 and instability index of 33.40. Molecular docking simulation of vaccine with TLR-5 with binding affinity of − 151.893 kcal/mol revealed good structural interaction and stability of protein structure of vaccine construct. The designed vaccine predicts the induction of immunity and boosted host's immune system through production of antibodies and cytokines, vital in hindering surface entry of parasites into host. This is a very important step in vaccine development though further experimental study is still required to validate these results.

## Introduction

The impact of coccidiosis on poultry production, and the evolution of drug resistance to known prophylactic drugs, attenuated and non-attenuated vaccines, is currently felt globally, and it is continuously inflicting grave economic loss to the poultry industry^1,2^. Avian coccidiosis is a ubiquitous intestinal disease caused by *Eimeria* species, which are intracellular obligate Apicomplexan protozoans^3–5^. This disease is characterised by the gut epithelial cells invasion by Eimerial sporozoites, resulting in clinical symptoms such as malabsorption and increased vulnerability to other pathogen infections^6,7^.

*Eimeria* parasites consist of fairly large genomes with their size ranging from 55 and 60 Mbp, carrying 8000 to 9000 genes^8^. The classification of *Eimeria* genomes includes the nuclear genome, which carries approximately 60 Mbp of DNA within 14 chromosomes of 1–7 Mb^5^. Other genomes are the mitochondrial genome of ~ 6200 bp, a circular apicoplast genome comprising ~ 35 kb circular extrachromosomal DNA and a double-stranded RNA viral genome^9^. This genome composition is the most complex and prone to evolve rapidly due to the fast life cycle of *Eimeria*^10^. *Eimeria* infection displays a direct faecal-oral life cycle, where the chickens ingest sporulated oocysts from polluted litter and feeds^11^. This infection can be visibly detected in chickens through clinical symptoms like intestinal inflammation, leading to haemorrhage and bloody diarrhoea. The clinical symptoms of this disease cause infected chickens to be vulnerable to other pathogens such as *Clostridium perfringens*, significantly compromising poultry's performance and productivity, resulting in high mortality^7,12^.

Over the years, the control of *Eimeria* in chicken production has heavily been dependant on the use of prophylactic chemotherapy and various vaccines containing components of live virulent or attenuated *Eimeria* as an effective mode of control^2^. However, the continuous administration of these anticoccidials has triggered drug resistance in parasites and compromised the quality of meat or egg production with traces of chemical residues detected in food, threatening food security^7,13^. Production of these vaccines and anticoccidial drugs require a high cost for their continued production^8^. This has urged the advancement of novel strategies to control and prevent the disease. The advancement in developing novel and cost-efficient vaccines against *Eimeria* is highly imperative to manage coccidiosis. The use of immunoinformatics on the existing knowledge about *Eimeria* species and the complexity of their life cycle has brought a paradigm shift in vaccine development, with most research shifting its focus to antigens involved in host/parasite interaction and in the developmental stages of the parasite.

Immune mapped protein-1 (IMP-1) is a membrane protein that is highly conserved, and a protective antigen discovered in *E. maxima*^13,14^. It was identified as an immunogenic and surface-expressed antigen that can confer protection against *Eimeria* infections and other apicomplexan parasites such as *Toxoplasma*^15^. IMP1 has also been identified as an immunoprotective antigen from other apicomplexan Eimeria species such as *E. tenella, E. mitis, E. acervulina* and *E necatrix*^16,17^. Various studies have shown that vaccination with formulations expressing IMP-1 from *Eimeria spp.* using different vectors as a delivery vehicle, induces significant protection against *Eimeria* challenge^13,18,19^. Literature has reported on the design and construction of multiepitope DNA vaccines and has confirmed the efficacy and safety of these vaccines against numerous pathogens such as *Plasmodium falciparum* (malaria)*, Bunyavirus (*Rift Valley fever virus*), Klebsiella Pneumoniae, Toxoplasma gondii* and *adenovirus serotype 4 (FAdV-4*)^20–23^. Construction of multiepitope vaccine also plays a crucial role in ongoing, ground-breaking research to find novel strategies to minimise the SARS-CoV-2 Corona Virus pandemic currently faced globally^24–27^. Hence this study explored *Eimeria* IMP-1 antigen with the aim to develop a potentially cost-effective multiepitope based vaccine (MEV) against *Eimeria* by identifying antigenic T-cell and B-cell epitopes through immunoinformatic techniques and evaluating the effectiveness of the identified epitopes in inducing immune response within the host through the construction of a multiepitope vaccine.

## Methods and materials

### The retrieval of Eimeria protein sequences and identification of conserved sequences from the genomic sequences

The protein sequences of *Eimeria* Immune mapped protein 1 (IMP1) antigen from different *Eimeria* species (see Supplementary Table S1) were retrieved in FASTA format from the protein database of the National Centre for Biotechnology Information- NCBI (https://www.ncbi.nlm.nih.gov/protein).

The obtained sequences were subjected to multiple sequence alignment (MSA) with conserved CD4 and CD8 chains capable of inducing immunological responses. MSA was performed using the CLUSTALW online server (http://www.genome.jp/tools-bin/clustalw)^28^. The MSA was performed using default parameters and obtained results of conserved regions that matched genome sequence and conserved chains, which had a minimum of 15 amino acid residues that were selected for further analysis.

### Antigenicity and prediction of transmembrane of conserved regions

In this study, the selected conserved regions were tested for antigenicity using the VaxiJen v2.0 Server (http://www.ddg-pharmfac.net/vaxijen/VaxiJen/VaxiJen.html)^29^, with the threshold set to 0.4. The sequences identified as probable antigens were selected and tested for transmembrane helix properties in the TMHMM v2.0 server (http://www.cbs.dtu.dk/services/TMHMM/). The TMHMM server was used to identify outer membrane protein sequences.

### Identification of T-cell epitopes

Since there is no data currently available in the immunoinformatic tool for chicken alleles used for MHC-epitope binding prediction, the human HLA alleles were selected and used as an alternative for the chicken MHC for both MHC-I and MHC-II epitope predictions^30,31^. As the chickens have different MHC alleles, studies have shown that anchor residues in chicken BF haplotypes are similar to residues anchored on mammalian MHC, especially those with 8–9mer in size, hence for this study MHC B locus was considered along with human alleles for the predictions^32^.

### Prediction of cytotoxic T-cell/CD8^+^ T-cell epitopes

The conserved sequences that fulfilled the transmembrane analysis were submitted to the NetCTL v1.2 tool to generate nonamers that can bind to major histocompatibility complex (MHC) class I HLA alleles molecules and induce CD8^+^. The resulting nonamers were subjected to the IEDB analysis tool (http://tools.iedb.org/mhci/) to determine cytotoxic T-cell (CTL) epitopes^33^. The nonamers were analysed using the Stabilized Matrix Base Method (SMM). The parameters for identifying MHC-I binding alleles selected included the amino acid length of peptide set to 9.0, the IC_50_ value < 250, and the human as MHC source species^21,34^. The generated epitopes were examined for antigenicity, with a threshold set to 0.5 as the main parameter. Sequences that were detected to be above the set threshold were selected as probable antigens. The CTL cell epitopes were further tested for immunogenicity using the MHC-I immunogenicity tool of IEDB (http://tools.iedb.org/immunogenicity/). The immunogenicity tool was mainly used to identify sequences that can stimulate an immune response towards any parasites in a host (human or animal)^20^.

### Helper T-cell/ CD4^+^ T-cell epitope prediction

The helper T-cell (HTL) epitopes were predicted using the IEDB MHC-II binding tool (http://tools.iedb.org/mhcii/). The prediction method used was SMM-align (stabilisation matrix alignment), allele length was set at 15, and the threshold for IC_50_ was < 250^35^. This technique also allowed the identification of MHC class II HLA alleles that bind to HTL epitopes. The identified HTL epitopes were subjected to the IFNepitope tool (http://crdd.osdd.net/raghava/ifnepitope/) to predict epitopes that could induce cytokine IFN-γ. The method and parameters used for prediction was the SVM (support vector machine) based method and the IFN-γ versus non-IFN-γ model. The identification of IL-4 inducers was achieved through the IL4pred tool (http://crdd.osdd.net/raghava/il4pred/). The resulting shortlisted epitopes were scrutinised using similar immunoinformatic tools as CTL epitopes, to determine their antigenicity with a threshold set at 0.5.

### B-cell epitope prediction

The B-cell epitopes were predicted using the antigenic and outer membrane protein sequences, initially identified in TMHMM and VaxiJen server. These sequences were input in the ABCpred server (http://osddlinux.osdd.net/raghava/abcpred/)^36^ to identify antigens that can induce antibodies and induce B-cell response. This server uses an artificial neural network to predict B-cell epitopes. The window length selected for prediction ranged from 12 to 16, with a threshold value of 0.51. The epitopes that overlapped with the final CD8 + T-cell epitopes and were observed to be non-allergenic were considered for the final vaccine construct.

### Conservancy and allergenicity test

The generated T-cell and B-cell epitopes identified as antigenic and immunogenic were tested for conservancy using IEDB conservation across antigen tool (http://tools.iedb.org/conservancy/)^37^. The allergenicity of the conserved epitopes was determined by the AllerTop v2.0 tool, where sequences identified as allergens were discarded, and only non-allergic sequences were selected^24^.

### Epitope merging for generation of multiepitope subunit vaccine

The prioritised epitope candidates for CD8^+^, CD4^+^ and B-cell epitopes were determined using various immunoinformatic tools and were joined together with an immunological adjuvant to form a multiepitope vaccine (MEV). The CD8^+^ T cell epitopes were joined together with the aid of AAY linkers, CD4^+^ T cell epitopes were linked by GPGPG linkers, and the B-cell epitopes were linked by the KK linkers. An appropriate adjuvant was attached to the N-terminal of the vaccine with the aid of the EAAK linker. These linkers provide extended flexibility to the peptides making up the vaccine, making it more stable. The addition of the adjuvant to the vaccine is crucial as it boosts the immunogenicity of the multiepitope construct^38^. The adjuvant added to the selected T & B-cell epitopes was a cholera toxin subunit B (CTB) sequence (accession no. ABV74245.1). CTB is the non-toxic component of the cholera toxin that forms pentamers with high binding affinity for the toxin's cell surface GM1-ganglioside receptor of the gut mucosa^39^. Gene expression profiling studies indicated that cholera toxin B stimulated TLR5 signalling pathway activation^40^.

### Antigenicity, allergenicity, solubility, and physiochemical properties assessment

The antigenicity of the final MEV sequence was tested using Vaxijen v2.0 server (http://www.ddgpharmfac.net/vaxijen/VaxiJen/VaxiJen.html). The predicted vaccine's antigenic nature ensured its ability to bind and interact with the receptor during the docking stage. AllerTop v2.0 was used to further determine the constructed vaccine as an allergen or non-allergen. To assess physicochemical parameters of the vaccine protein, the vaccine sequence was subjected to ProtParam53 web server (https://web.expasy.org/cgi-bin/protparam/protparam/) from the Expert Protein Analysis System (EXPASY) to calculate the number of amino acids of the vaccine, molecular weight (kDa), theoretical isoelectric point (pI), estimated half-life, instability index, aliphatic index, hydropathicity GRAVY^41^.

### Tertiary structure prediction, refinement, and validation

The tertiary structure of the previously designed MEV protein was predicted and generated using the RaptorX server (http://raptorx.uchicago.edu/). Since the designed vaccine was a novel protein without any known template, Raptor was the best suited for structural predictions. Molecular refinement of the predicted vaccine tertiary structure was achieved using the GalaxyRefine server (http://galaxy.seoklab.org/cgi-bin/submit.cgi?type=REFINE)^27,42^. The structure refinement was done to improve the structural quality of the vaccine protein. The GalaxyRefine server predicted five refined models of the vaccine construct resulting from structural perturbations and structural relaxations. From the refined models, model 1 was predicted by structure perturbation applied to the clusters of the side chains and models 2–5 were generated by more aggressive perturbations^43^. All the five refined models were further checked for GDT-HA, RMSD, MolProbity score and the best-refined model was selected and validated. The validation of the selected refined tertiary structure for the designed vaccine protein was performed using PROCHECK, which generated a Ramachandran plot, and ProSA-web (https://prosa.services.came.sbg.ac.at/prosa.php) was employed for final validation which generated a Z-score for confirmation^44^.

### Molecular docking of vaccine constructs with Toll-like receptor 5

Docking of vaccine with TLR5 (PDB ID: 3V44) was done to check the vaccine’s binding affinity and agonistic ability towards receptor molecule. The TLR5 was selected as a receptor to bind the designed vaccine because it was reported to be ortholog of TLRs found in humans and it was highly expressed in cecum primary immune effector cells of infected chickens with mature natural flora^45,46^. To start docking, solvent accessibility was calculated with the Naccess tool (http://wolf.bms.umist.ac.uk/naccess/) to access the active and passive residues of both the vaccine construct and TLR5, which were then used in docking simulation. The PDB structure of TLR5, active residues of receptor and sequence of the MEV were submitted to AttractPep (http://www.attract.ph.tum.de/services/ATTRACT/peptide.htm) ^47^ and completed using a locally installed attract package on the Centre of High-Performance Computing (CHPC) Lengau cluster. A total of 50 models were generated for the MEV_TLR5 complex. The model with the lowest energy and binding properly to the receptor was selected and visualised using VMD 1.9.3 software (http://www.ks.uiuc.edu/Research/vmd/)^48^. The UCSF-Chimera v1.14 software (http://www.cgl.ucsf.edu/chimera/) ^49^ was later used to visualise the best-docked tertiary structure of the refined TLR-vaccine.

### Molecular dynamics simulations and analysis

Docked complex of MEV and TLR5 was further subjected to energy minimisation through molecular dynamics simulations (MDS) using AMBERS 14 package^50^. MDS was performed to evaluate complex stability and interactions between the docked proteins^51^. The input proteins were described using FF14SB^52^, and topologies of the vaccine structure were generated using the LEAP module of AMBER 14. This was done by introducing ions (protons and Cl-) into the orthorhombic solvation box of TIP3P water molecules of 8 Å, to neutralise the system^53^. To minimise high energy configurations in the protein, energy minimisation was performed to obtain the lowest energy of the protein^54^. This step was initially performed with 10,000 steps (500 steepest descents with 9500 conjugate gradient) and followed by full minimisation of 2000 steps. The system was gradually heated for 2 ns (ns) in a canonical ensemble (NVT) with a Langevin thermostat (from 0 to 300 K). The collision frequency applied to the system was 1.0 p s^−1^, with the density of the water system regulated with 4 ns of NPT simulation. The molecular dynamic production was run for 100 ns of NPT (constant number N, pressure P and temperature T), where equilibration of the entire system was reached at 300 K for another 2 ns at pressure of 1 bar. After molecular dynamic simulation, the PTRAJ and CPPTRAJ modules in AMBER 14 were used to analyse parameters: Root Mean Square Deviation (RMSD) and Root Mean Square Fluctuations (RMSF).

### *In silico* codon optimisation, cloning and expression of vaccine construct

Codon optimisation of the multiepitope construct was achieved using Java Codon Adaptation Tool (JCat:http://www.jcat.de/)^55^. The optimisation was performed to obtain an improved nucleotide sequence adapted to its potential selected expression host (*E. coli* strain K12). The JCat adaptation is dependent on the codon adaptation index (CAI) and percentage GC content of improved sequence. The optimal CAI score of optimised gene sequence ranges from is 0.8 to 1.0 and GC% (30–70%), indicating improved expression of a gene in its corresponding organism, without any translation errors^51^. The optimised nucleotide sequence of the vaccine was cloned and expressed in *E. coli* (strain K12) host, where XhoI (CTCGAG) and BamHI (GGATCC) restriction sites were added to 5` and 3` ends of the construct prior to cloning, respectively. To clone the improved vaccine sequence into a suitable expression vector, SnapGene viewer v5.3 software (http://www.snapgene.com/) was used.

### Immune simulation

The multiepitope peptide was subjected to an online *in silico* immune simulation server (C-ImmSim: http://kraken.iac.rm.cnr.it/C-IMMSIM/) to generate and evaluate vaccine candidate immune response^56^. All simulation parameters used for simulation were set at default, where a single injection and the vaccine with no lipopolysaccharide (LPS) option were selected. The vaccine was administered at 3 intervals of 4 weeks.

## Results

### The retrieval of Eimeria protein sequences and identification of conserved sequences from the genomic sequences

To design the multiepitope subunit vaccine, a total of 19 *Eimeria* Immune Mapped protein-1 antigen genomic sequences representing all *Eimeria* species were retrieved from NCBI. The genome's conserved sequences were created through multiple sequence alignment using the CLUSTALW online server, where 22 unique and conserved sequences were selected.

### Antigenicity and prediction of transmembrane of conserved regions

All the selected conserved sequences were tested for antigenicity with default parameters of the threshold value set at ≥ 0.4, in Vaxijen v2.0 server. It was found that 16 sequences fulfilled the antigenicity property of set threshold with Vaxijen score ranging from 0.4295 to 0.9046 (Table 1). The transmembrane analysis performed using TMHMM v2.0 server detected about 12 conserved sequences that fulfilled and exhibited the exomembrane properties (Table 1).

**Table 1:** Conserved sequences of the genome sequence of *Eimeria*.

Conserved sequences	Antigenicity score	Transmembrane helix
AEEIENKVLPVKEEDAFNISAFGFVAVTPPPPPYKAGANITPKRFGEIATGAGGAYLQLS	0.4295	Outside
GITYFLQEMKYKWEVWSKVQRQAYYQGWIKFVKAADEMEASFTLHHFAAPAPPAKLFLLH	0.5288	Outside
GKNELIRNLQSDKKLFYSGICQFVKEAKDIKGKLTLLQHFDSSFPIKVDLYF	0.5931	Outside
GVTCLLQEMKYKWDVWSKVQRQPYYQGWMKFIKAADEMEASVKVHQFTSPAPAAKVFLLH	0.5782	Outside
MGAACGKSQRAAAAVEPPLSTAEKAEAAAVAAAEHSQKAEEAAEVAAACATK	0.7171	Inside
MGAACMKSHGAATDAVAPRRSTAEKAADAAAAAEEHSHKAQEAAETAAACARR	0.7931	Inside
MGAACMKSHIGPRSAAEAACPPTAAEKAAEAASEAAIHSGKPEEAEEAAAAAAEAPGAAV	0.6764	Outside
MGAACMKSQGGATPPAAGGVSAAQKVAEATAAAAEHSMKAHQAAETAAACAKR	0.7150	Inside
MGAACTKSKGTAAPAARPSTTDRATEAAAAAAEHSQKAQEAAEEAIACAAK	0.7889	Inside
MGGACGKSRGTAAAAAAAPPVSAADKAAEAAASAASHAEKAQEAAAAAAAAAAN	0.9042	Outside
MGGACGKSRGTAAAAEPPVSAADKAAEAAASAASQAEKAQEAAAAAAAAAAN	0.9046	Outside
SGPIENKVLPVMEEESFSVSVFGFAAVTPPPSPYKAGANISPKRFGEIATAAGGGYVQLS	0.6084	Outside
TGPGGPTYSFLAEGGMLHLMKPRCFCLMFLALKWD	0.6811	Outside
TGPIENKVLPAKEEEPFNVSVFGLAAVTPPSPPYKPGANITPKRFGEIATGAGGAYMQLS	0.5831	Outside
TGPIENKVLPVKEEEEFKISVFGFAAVVPAQSSYKPGANITPKRFGEIATEAGGAYIQLS	0.7513	Outside
TGPIENKVVPVKLGEPIGISMFGFAAVAPPPAPYKAGANITPKRFGELATQAGGAYIQLS	0.4525	Outside

### Prediction of T-cell epitopes

#### Prediction of cytotoxic T-cell/CD8^+^ T-cell epitopes

The conserved sequences selected were subjected to the NetCTL v1.2 server, where a total of 577 receptor-specific immunogenic nonamers of CTL epitopes were found. The nonamers were subjected to the IEDB MHC-I prediction tool, where the SSM-based method and the IC_50_ value parameter < 250 were set to predict MHC-I binding alleles accurately. The prediction analysis detected that about 214 CTL epitopes interacted with one to eight MHC alleles under the set IC_50_ parameter. The selected epitopes were further tested for antigenicity with a threshold value set at ≥ 0.5, in Vaxijen v2.0 server. A total of 77 CTL epitopes were detected to be antigenic in nature, with epitope ‘FKISVFGFA’ having the highest Vaxijen score of 2.2931. The immunogenicity analysis performed using the IEDB tool identified 41 sequences as immunogenic. These epitopes were tested for the conservancy, where 20 epitopes were noted to be conserved. The conserved epitopes also underwent allergenicity analysis using AllerTop v2.0 server, where seven (7) CD8^+^ epitopes: AQEAAAAAA, EAAAAAAAA, FGFVAVTPP, FKISVFGFA, FNISAFGFV, FTSPAPAAK and KISVFGFAA were found to be non-allergens and were regarded as final predicted epitopes. The summary of results obtained for the final predicted CD8^+^ T cell epitopes, IC_50_, antigenicity, immunogenicity and allergenicity scores are presented in Table 2.

**Table 2:** Final predicted CD 8^+^ T cells epitopes, 100 overlapped with CD4^+^T cell epitopes and interacting with different MHC-I alleles.

Epitopes	HLA alleles	IC_50_	Antigenicity	Allergenicity
AQEAAAAAA	HLA-A*02:06	137.26	0.8280	Non-allergen
EAAAAAAAA	HLA-A*35:01	124.33	0.8030	Non-allergen
	HLA-A*68:02	34.087		
FGFVAVTPP	HLA-A*02:06	199.779	0.6274	Non-allergen
FKISVFGFA	HLA-A*02:06	139.49	2.2931	Non-allergen
FNISAFGFV	HLA-A*68:02	34.80	1.4278	Non-allergen
	HLA-A*02:06	57.22		
	HLA-A*02:03	227.48		
FTSPAPAAK	HLA-A*68:01	35.11	1.0977	Non-allergen
	HLA-A*11:01	158.23		
KISVFGFAA	HLA-A*02:06	228.32	1.4171	Non-allergen

#### Prediction of helper T-cell/ CD4^+^ (HTL) epitopes

The 12 conserved sequences that previously fulfilled the transmembrane analysis were subjected to the IEDB MHC-II binding tool to predict HTL epitopes and their respective HLA alleles. The VaxiJen server was applied to the detected HTL epitopes to test for antigenicity. A total of 103 epitopes were obtained that fulfilled both the IEDB tool and Vaxijen parameters of IC_50_ value < 250 and ≥ 0.5 antigenicity score were considered as potential HTL epitopes. The identified HTL epitopes were assessed for the conservancy, where 45 CD4^+^ T- cell epitopes were selected as conserved. The identified conserved epitopes were further subjected to IFNepitope and IL-4pred immunoinformatic tools to identify HTL epitopes that can induce immune response by producing signal cytokines, i.e., IFN-gamma inducers and interleukin inducers. A total of 25 CD4^+^ T-cell epitopes exhibited both IFN-gamma and IL-4 inducer properties, enhancing the immunogenic capacity of the potential vaccine. These epitopes were subjected to AllerTop v2.0 for allergenicity analysis, where the allergenicity analysis revealed 21 epitopes to be non-allergenic, making them final suitable predicted HTL epitopes candidates for vaccine development since they do not cause any allergic reactions to the host (Table 3). The epitope ‘KEEEEFKISVFGFAA’ with the highest antigenic VaxiJen score of 1.5250 was disregarded as the allergenicity analysis identified it as allergic.

**Table 3:** Final predicted CD4^+^ T-cells epitopes, 100 overlapped with CD8^+^ T cell epitopes and interacting with different MHC-II alleles.

Epitopes	HLA alleles	IC_50_	Antigenicity	Allergenicity
KAQEAAAAAAAAAAN	HLA-DRB1*01:01	235.00	0.8516	Non-allergen
AEKAQEAAAAAAAAA	HLA-DRB1*01:01	232.00	0.8224	Non-allergen
EKAQEAAAAAAAAAA	HLA-DRB1*01:01	230.00	0.8188	Non-allergen
AFNISAFGFVAVTPP	HLA-DRB1*04:04	248.00	0.9478	Non-allergen
AYYQGWIKFVKAADE	HLA-DRB1*04:04	62.00	0.6862	Non-allergen
	HLA-DRB1*04:05	119.00		
DAFNISAFGFVAVTP	HLA-DRB1*09:01	155.00	0.7499	Non-allergen
	HLA-DRB1*01:01	221.00		
EDAFNISAFGFVAVT	HLA-DRB1*09:01	152.00	0.6700	Non-allergen
	HLA-DRB1*01:01	204.00		
EEDAFNISAFGFVAV	HLA-DRB1*09:01	154.00	0.5405	Non-allergen
	HLA-DRB1*01:01	209.00		
EEEEFKISVFGFAAV	HLA-DRB1*09:01	234.00	1.1886	Non-allergen
	HLA-DRB1*01:01	194.00		
EEEFKISVFGFAAVV	HLA-DRB1*09:01	214.00	1.3741	Non-allergen
	HLA-DRB1*01:01	162.00		
EFKISVFGFAAVVPA	HLA-DRB1*01:01	112.00	1.5196	Non-allergen
FKISVFGFAAVVPAQ	HLA-DRB1*01:01	138.00	1.3738	Non-allergen
FTLHHFAAPAPPAKL	HLA-DRB1*04:04	87.00	0.6040	Non-allergen
	HLA-DRB1*09:01	248.00		
	HLA-DRB5*01:01	151.00		
	HLA-DRB1*01:01	86.00		
KEEDAFNISAFGFVA	HLA-DRB1*09:01	172.00	0.7168	Non-allergen
KISVFGFAAVVPAQS	HLA-DRB1*08:02	118.00	0.8556	Non-allergen
	HLA-DRB1*09:01	45.00		
	HLA-DRB1*04:04	46.00		
	HLA-DRB1*04:05	134.00		
	HLA-DRB1*04:01	183.00		
	HLA-DRB1*01:01	32.00		
	HLA-DRB5*01:01	108.00		
	HLA-DRB1*07:01	218.00		
QAYYQGWIKFVKAAD	HLA-DRB1*04:05	142.00	0.8529	Non-allergen
SFTLHHFAAPAPPAK	HLA-DRB1*04:04	86.00	0.6518	Non-allergen
	HLA-DRB5*01:01	165.00		
	HLA-DRB1*01:01	92.00		
TLHHFAAPAPPAKLF	HLA-DRB5*01:01	147.00	0.5433	Non-allergen
	HLA-DRB1*04:04	229.00		
	HLA-DRB1*01:01	95.00		
VHQFTSPAPAAKVFL	HLA-DRB5*01:01	28.00	0.7514	Non-allergen
	HLA-DRB1*01:01	104.00		
VKVHQFTSPAPAAKV	HLA-DRB5*01:01	31.00	0.9198	Non-allergen
	HLA-DRB1*09:01	225.00		
	HLA-DRB1*04:04	153.00		
	HLA-DRB1*01:01	106.00		
YYQGWIKFVKAADEM	HLA-DRB1*04:04	60.00	0.5970	Non-allergen
	HLA-DRB1*04:05	116.00		

### B-cell prediction

The B-cell epitopes were predicted using the ABCpred server, where 12 conserved genomic sequences that passed the transmembrane property test were used as input template. A total of 71 B-cell epitopes were generated and further tested for antigenicity, allergenicity and conservancy. From the generated epitopes, only 6 B-cell epitopes (Table 4) passed the criteria mentioned above.

**Table 4:** Final predicted B-cell epitopes with their antigenicity and allergenicity.

B-cell epitopes	ABCpred score	Position	VaxiJen score	Conservancy	Allergenicity
ASHAEKAQEAAAAAAA	0.73	327	0.9352	100%	Non-allergen
ASQAEKAQEAAAAAAA	0.65	379	0.8808	100%	Non-allergen
EEEFKISVFGFAAVVP	0.71	567	1.4763	100%	Non-allergen
KVHQFTSPAPAAKVFL	0.87	215	0.8546	100%	Non-allergen
QEAAAAAAAAAANSGP	0.67	386	0.8157	100%	Non-allergen
VKEEDAFNISAFGFVA	0.64	11	0.6457	100%	Non-allergen

### Epitope merging for generation of multiepitope subunit vaccine

The multiepitope vaccine (MEV) candidate against *Eimeria* was constructed by joining together the prioritised final predicted 7 CTL epitopes, 21 HTL epitopes and 6 B-cell epitopes using AAY, GPGPG and KK linkers and the adjuvant was attached at the N-terminal and appropriate linkers to enhance the immunogenicity of the construct. This resulted in the multiepitope vaccine containing 731 amino acid residues, which was further validated for antigenicity, allergenicity and physicochemical properties.

### Antigenicity, allergenicity, solubility, and physicochemical properties assessment

The constructed MEV was subjected to various tools to validate its effectiveness as a vaccine. The vaccine was found to be antigenic with Vaxijen score of 0.6043, non-allergic and immunogenic (score = 8.59968), making it a potentially good candidate to provoke an effective immune response in the host. The physiochemical properties evaluated using the ProtParam server revealed that the designed multiepitope vaccine had a molecular weight of 73.25 kDa, theoretical pI of 8.23 and instability index of 33.40. The obtained theoretical pI and instability index classified the vaccine protein as acidic and stable. The half-life of the vaccine was assessed to be 1 h (h) (in vitro) in mammalian reticulocytes 30 min in yeast and > 10 h in *E. coli,* in vivo. The aliphatic index and grand average of hydropathicity (GRAVY) were 66.46 and 0.140, respectively^48^. The obtained aliphatic index and GRAVY suggests the designed vaccine is thermostable and hydrophobic in nature. The overall physiochemical properties and allergenicity results revealed that the multiepitope vaccine is immunogenic, non-allergenic and thermostable, making it appropriate for vaccine production^16^.

### Tertiary structure prediction, refinement, and validation

**Figure 1:**
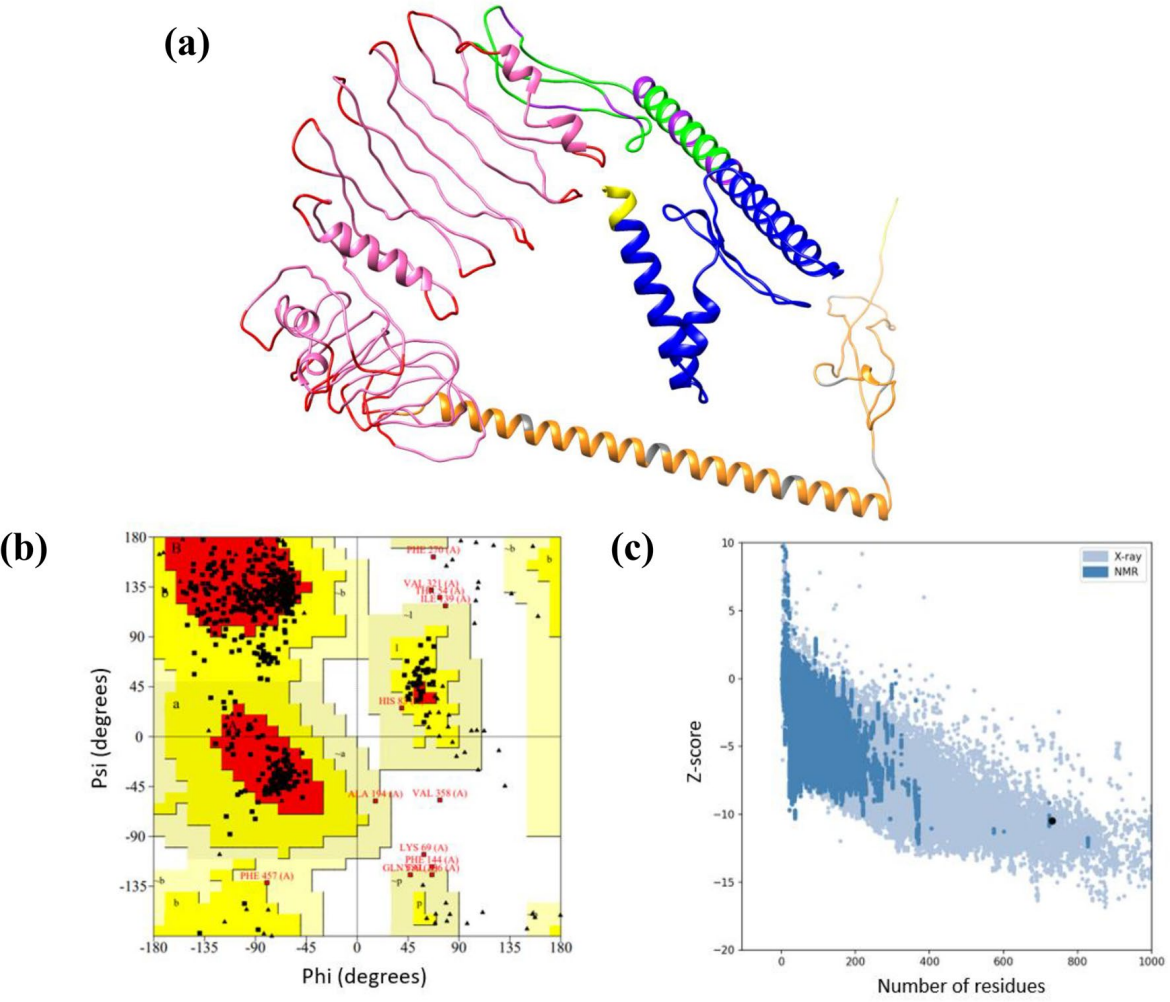
Vaccine construct structural validation analysis (**a**) Refined model of multiepitope vaccine construct (CD8^+^ T-cell epitopes and AAY linkers-green and purple, CD4^+^ T-cell epitopes and GPGPG linker—pink and red, B-cell epitopes and KK linkers-orange and gray and the adjuvant—blue) (**b**) Ramachandran plot of refined vaccine (83.2% of the residues of the vaccine are present in the favoured region (**c**) PROSA web score plot indicating a Z-score =  − 10.48, indicating overall model quality.

**Figure 2:**
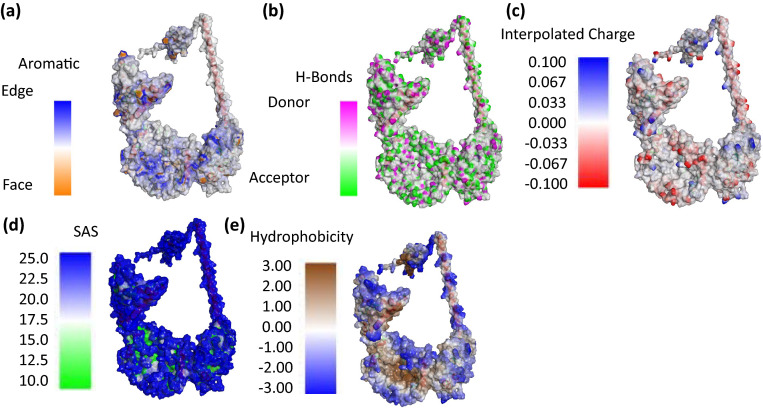
The structural quality of the vaccine construct (**a**) Aromatic amino acid residues (**b**) hydrogen bonds with donor acceptor atoms (**c**) charge distribution with the red, white and blue indicated negative, neutral and positive charged residues respectively (**d**) solvent accessibility surface from blue for exposed to green for buried and (**e**) the hydrophobic surface of the MEV with red and blue representing hydrophobicity and hydrophilicity respectively.

The tertiary structure of the designed multiepitope vaccine was predicted using the RaptorX server. Since the designed vaccine had no structural template, the RaptorX server was the suitable platform to generate the vaccine's tertiary structure. The server produced five different potential models which were validated using the PROCHECKER server. Model 4 was chosen from the models produced as potential vaccine structure with Ramachandran plot percent of 79.8% of residues in the favoured region. However, the validation showed that the designed vaccine peptide had some missing residues. Hence, the selected model was refined using GalaxyRefine server. The server also produced five potential vaccine models with an improved number of residues allowed in the favoured region. The best model selected was Model 5 (Fig. 1a), which showed an improved percentage of 83.2% in the Ramachandran plot analysis when revalidated after refinement (Fig. 1b). Other favourable parameters obtained for the refined model included: GDT score of 0.8684, RMSD value of 0.626, MolProbability of 3.399, clash score of 84.1, and poor rotamers of 3.8. The selected model was further validated using ProSA- web, where a Z-score of − 10.48. The Z-score obtained suggests that the designed vaccine is of good quality since it lies within the vicinity of PBD X-ray experimental structures (Fig. 1c). The structural quality of the vaccine construct in addition to the physicochemical properties previously discussed are displayed in Fig. 2.

### Docking and Molecular dynamic simulation of vaccine constructs with Toll-like receptor 5

**Figure 3:**
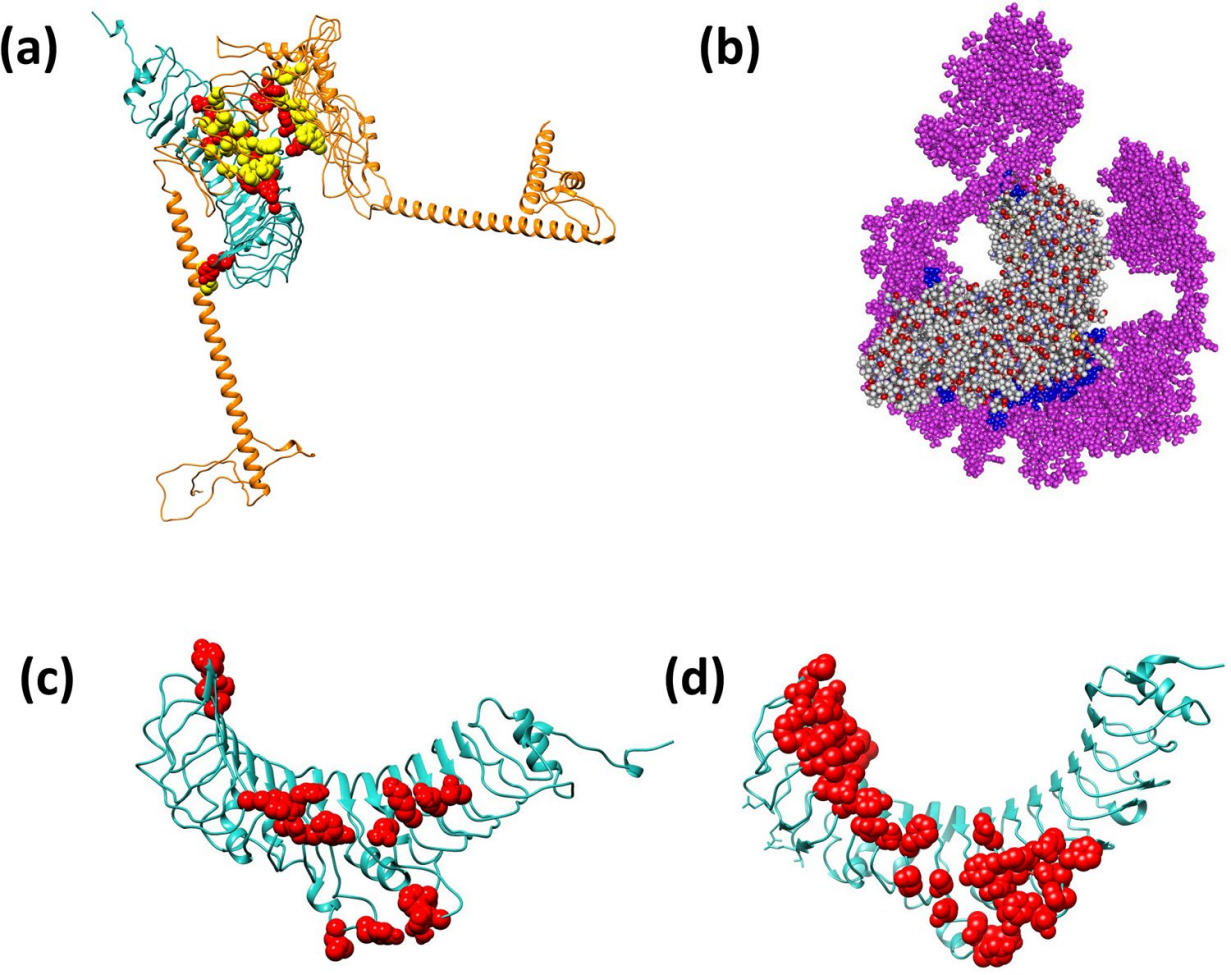
(**a**) Molecular interaction 3D structure of the designed MEV (orange) with TLR5 (light green) after docking analysis where interacting residues indicated by red (TLR5) and yellow (vaccine), (b) the complex after MD simulation with MEV (purple), TLR5 (multi-colour) and interacting residues (blue), (**c**) the mapped binding domain of MEV on TLR5 and (**d**) the mapped binding domain of *Salmonella* flagellin on TLR5.

**Figure 4:**
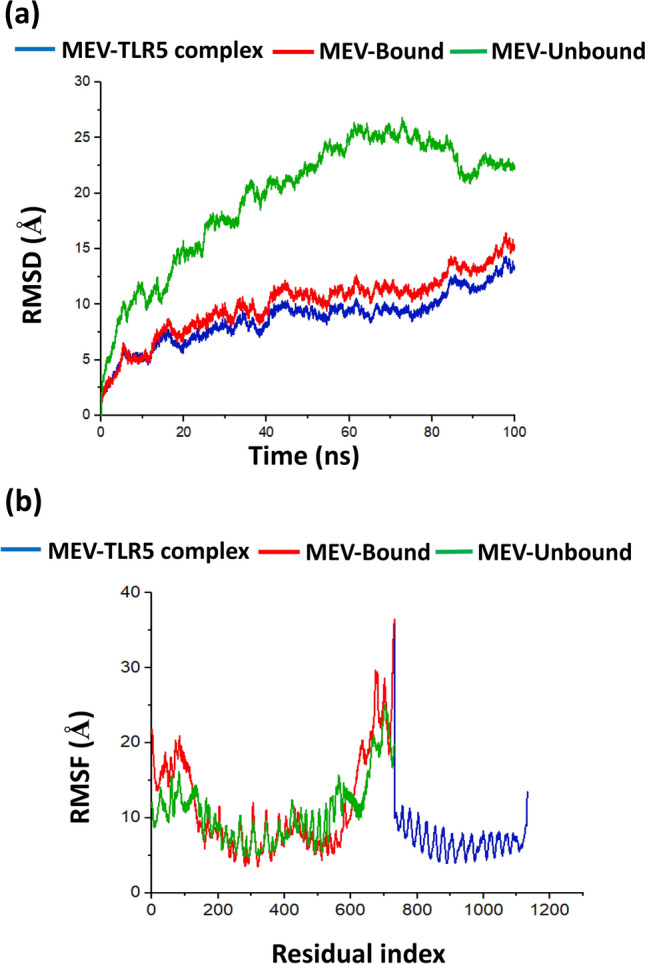
Molecular dynamics simulation of MEV-TLR5 docked complex, MEV bound with TLR5 and unbound MEV (not docked with TLR 5) at 100 ns time duration (**a**) RMSD plots showing structural stability through analysis of amino acid backbone. The MEV binding to TLR5 (red plot) showed more stability than the unbound MEV (green plot) and (**b**) RMSF plot showing interaction of side chain atoms.

The docking of the vaccine with receptor TLR5 (PDB ID: 3V44) produced 50 docked complexes and the produced models were visualised using the VMD software and Chimera v2.0 to observe the vaccine's interaction and binding to the TLR5 complex. The best-docked complex was selected with a binding affinity of − 151.893 kcal/mol (Fig. 3a). Figure 3c showed that the MEV from the present study bound to the binding domain of TLR5 when compared to the binding domain of *Salmonella* flagellin mapped on the TLR5 (PDB ID: 3V47) in Fig. 3d, which was an indication of good interaction of the MEV with the TLR5. Molecular dynamics (MD) simulation (Fig. 3b) was performed to assess the stability and binding of the docked complex using parameters RMSD and RMSF. The structural flexibility of the MEV_TLR5 complex after MD simulation was demonstrated by RMSD results (Fig. 4a), where fluctuation for backbone atoms of MEV before docking were within 1.5 Å to 25.0 Å whereas the fluctuation of the complex after MD simulation was within 1.5 Å to 15.0 Å. The binding of MEV to the TLR5 caused more stability to the MEV system as displayed in the figure. RMSF analysis (Fig. 4b) revealed side chain atoms of docked complex that exhibited high interaction between vaccine and TLR5, with fluctuation regions at 50–180 and 510–660 residues. The binding energy recorded from the MD simulation was − 47.14 ± 0.46 kcal/mol with contributions from other component energy such as van der Waal (− 167.30 ± 0.54 kcal/mol), gas phase energy (− 665.32 ± 3.50 kcal/mol), electrostatic (− 498.02 ± 0.54 kcal/mol), electrostatic contribution to the solvation of free energy (639.55 ± 3.23 kcal/mol), non-polar solvation energy (− 21.37 ± 0.08 kcal/mol) and solvation free energy (618.18 ± 3.16 kcal/mol). Energy decomposition of the interacting residues of both TLR5 and MEV with cut off ≤  − 1.0 is within the range of − 6.106 to − 1.222 kcal/mol as presented in Table 5.

**Table 5:** Energy decomposition of the interacting residues of MEV_TLR5 complex during molecular dynamic simulation.

Interacting MEV residue	Decomposition energy (kcal/mol)	Interacting TLR5 residue	Decomposition energy (kcal/mol)
PHE 316	− 6.106 ± 0.597	LEU 925	− 4.072 ± 0.849
PHE 296	− 4.773 ± 1.069	HIE 983	− 3.799 ± 1.021
PHE 277	− 2.653 ± 0.440	GLN 918	− 2.830 ± 2.132
TRP 232	− 2.426 ± 0.773	TYR 923	− 2.512 ± 0.928
PHE 337	− 2.087 ± 0.709	PHE 986	− 2.122 ± 0.388
PRO 283	− 2.064 ± 1.664	TYR 738	− 1.703 ± 1.380
GLY 336	− 1.999 ± 0.351	LYS1011	1.529 ± 0.283
PHE 335	− 1.944 ± 0.721	GLY 982	− 1.458 ± 0.699
VAL 547	− 1.756 ± 1.122	ASN 758	− 1.349 ± 1.904
GLY 317	− 1.727 ± 0.494	LYS1009	1.332 ± 0.579
PRO 565	− 1.687 ± 1.457	ASN 985	− 1.258 ± 0.541
PRO 465	− 1.570 ± 1.077	LYS 867	− 1.222 ± 2.212
PHE 508	− 1.467 ± 0.560		
PRO 505	− 1.343 ± 0.786		
ALA 279	− 1.297 ± 0.631		
ALA 616	− 1.281 ± 1.121		
LYS 234	1.232 ± 1.071		
VAL 280	− 1.178 ± 0.822		
ILE 468	− 1.151 ± 0.563		
ASP 247	− 1.136 ± 2.295		
PRO 485	− 1.006 ± 0.582		

### *In silico* codon optimisation, cloning and expression of vaccine construct

**Figure 5:**
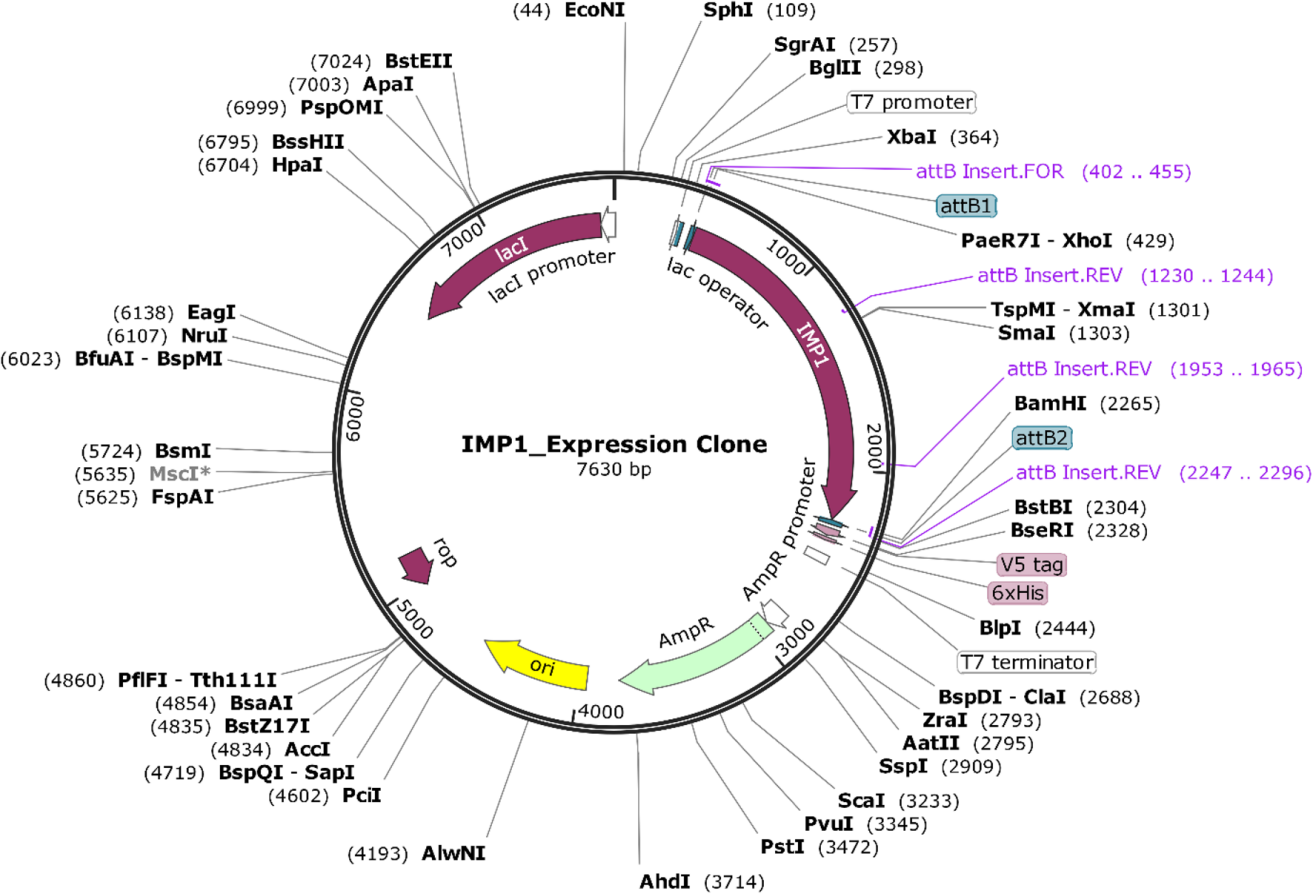
*In silico *cloning map for improved multiepitope sequence into petDEST42 vector, showing multiepitope vaccine labelled as IMP1 connected with XhoI (429) at C terminal and BamHI (2265) N terminal.

To optimise the use of codons of the vaccine structure for maximal expression in *E. coli* (strain K12), the Java Codon Adaptation Tool (JCat) was used. The improved nucleotide sequence of the vaccine was found to have CAI value of 1.0 and GC-content of 55.19, showing a high probability of expression of the vaccine in the *E. coli* K12 strain. Finally, *in silico* cloning of the improved sequence was performed using SnapGene software, where XhoI and BamHI restriction sites were added into the final vaccine and closed into the expression vector petDEST42 (7630 bp construct) (Fig. 5).

### Immune simulations for vaccine construct

**Figure 6:**
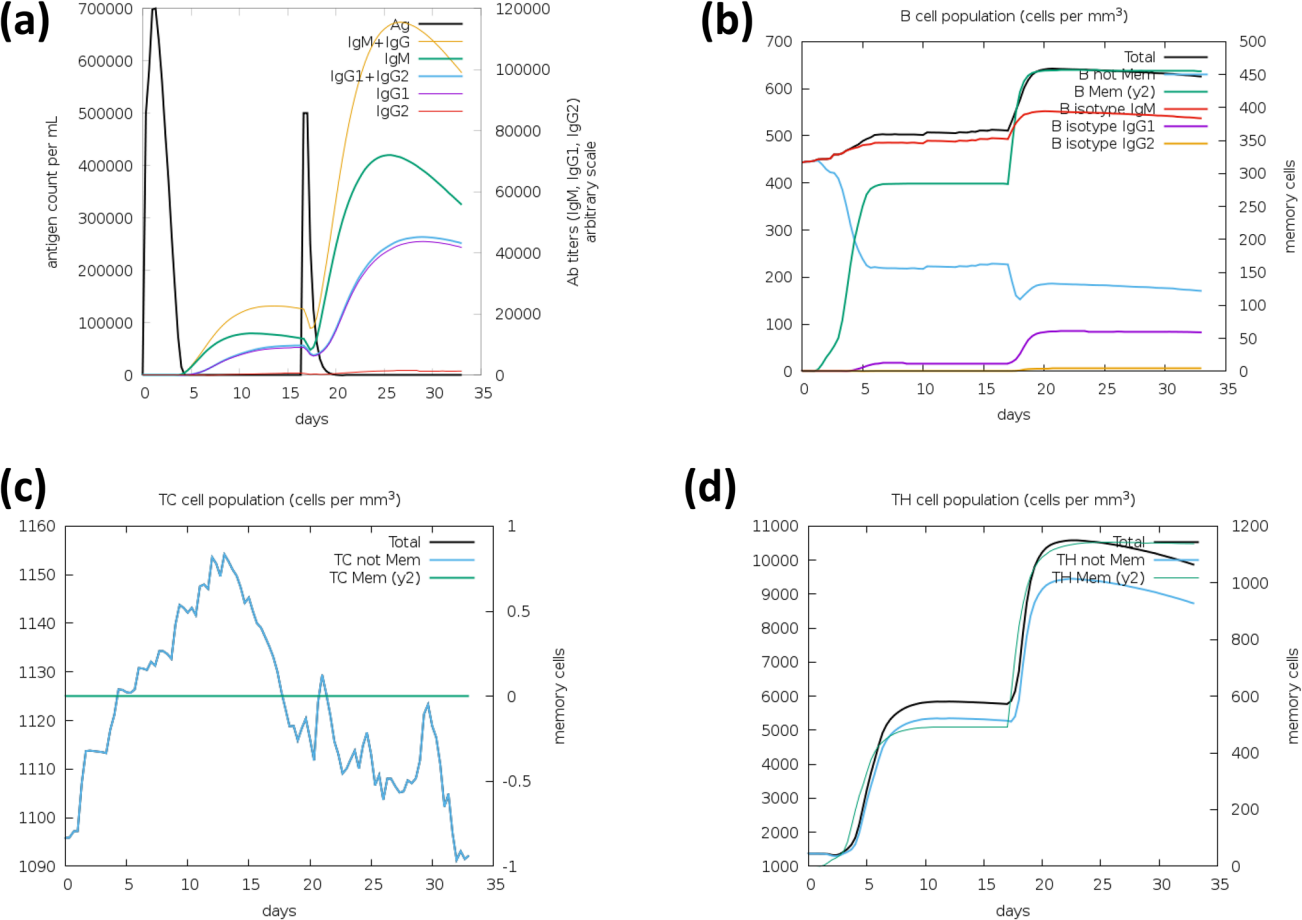
Immune simulation results from C-IMMSIM of the vaccine construct showing immune response through (**a**) production of immunoglobulin in response to antigen injections (black vertical lines) and (**b**) B lymphocytes: total count, memory cells, and sub-divided in isotypes IgM, IgG1 and IgG2 (**c**) CD8 + T-cytotoxic lymphocytes count (**d**) CD4 + T-helper lymphocytes count.

The results of immune simulation revealed different immune profiles produced by the vaccine, where the vaccine was observed to induce an immune response by the increase of antibodies upon administration to the simulation. The administration of the vaccine candidate showed a significant increase in tertiary immune response exhibited by high levels of IgG1 + IgG2, IgG + IgM, IgG1 and IgG2 compared to the primary response represented by IgM (see Supplementary Fig. S1a). Exposure of the B-cell population to the vaccine showed constant increased levels, developing memory cells to keep the vaccine's memory if the host encounters reinfection (Fig. 6b). The subsequent injection of vaccine construct also exhibited a significant increase in cytokines such as IFN-gamma, TGF-b, IL-10, IL-23, and IL-12, indicating a good immune response (see Supplementary Fig. S1a), correlating with the prediction of IFN-gamma epitopes in the vaccine. The decline in antigen level with each injection confirmed the presence of antibodies that effectively maintained the possibility of an antigenic surge.

## Discussion

Coccidiosis is a ubiquitous disease caused by *Eimeria* in livestock^7,57,58^. This disease has caused substantial economic loss globally in the chicken industry due to the high mortality of chickens leading to reduced productivity. Vaccination of poultry at an early age is crucial to curb the economic impact that coccidiosis currently pose to the poultry industry. Due to their rapid life cycle, consisting of multiple stages within a host, the complexity of *Eimeria* infections requires novel approaches in vaccine design to improve their efficacy. This study aimed to apply immunoinformatic and reverse vaccinology to generate a multiepitope vaccine candidate capable of inducing a targeted response against *Eimeria*.

Advances in the immunoinformatic approach enables the prediction and selection of specific immunogenic machinery of the parasite responsible for the disease rather than the inclusion of the whole cell, preventing undesirable immune response which may result in enhanced allergenicity of the parasite towards a host, reducing the time required for the design of the vaccine^22,23,39^. The tools involved in epitope prediction are critically selected based on their accuracy to ensure the design of long-lasting immunogenic vaccines with high efficacy. The T-cell and B-cell epitope prediction is achieved through IEDB servers and ABCpred server, respectively. Several recent studies have implemented these tools to predict epitopes and develop multiepitope vaccines^17,34,36,51,59^. Even though most prediction tools used to identify B-cells have low accuracy, ABCpred has been considered one of the most effective epitope prediction servers available with an accuracy of 65.93%^26^. To ensure the uniqueness and efficiency of epitopes identified with the tools mentioned above, further analysis (such as antigenicity- efficiency = 70–80%, allergenicity, conservancy, etc.) of the predicted epitopes is required. While the accuracy of these tools ranges from ~ 65 to 88% which is well above average, they produce predictive results that need further justification in the laboratory. Similar tools were used in the present study to predict and design a multiepitope vaccine.

In this study, the genomic sequence of *Eimeria* IMP-1 antigen was exploited to predict and design an immunogenic multiepitope vaccine candidate. The inclusion of different epitopes in the vaccine design may potentially provide maximum protection against various *Eimeria* spp. strains. Song et al.^2^ suggested that merging T-cell epitopes from different stages of the *Eimeria* life cycle could overcome the parasite's antigen complexity, which this study aimed to explore through designing a multiepitope vaccine. An effective vaccine should stimulate T (CTL or HTL) cells and B-cells capable of inducing immune responses to eliminate any pathogenic antigens within a host. To achieve this, the conserved sequences of IMP-1 antigen were selected and used to generate 28T-cell epitopes (7 CTL and 21 HTL) and 6 B-cell epitopes that were 100% conversed, antigenic and immunogenic vaccine candidates. The use of T- and B cells capable of eliciting a strong humoral and innate response in the vaccine construct ensured that the vaccine designed would confer double protection against the parasite^54^. The CD4^+^ helper T cells were further screened for IFN-γ and IL-4 inducing properties. The presence of these inducers in a vaccine construct is crucial for initiating the production of cytokines that activate an immune-stimulatory response through the production of cytokines (danger signalling cells). Tang et al.^13^ reported that IFN-γ and IL2 could elicit lymphocytes and are involved in Th1 mediated immune response and Th2 immune response, respectively. Hence the inclusion of epitopes capable of inducing cytokines was crucial for improving the efficacy of the vaccine. The final predicted T-cells (7 CTL epitopes and 21 HTL epitopes) and 6 B-cell epitopes were joined together by flexible linkers to form the vaccine construct with 731 aa. An ideal vaccine also depends on the effectiveness of the adjuvant selected to improve the host immune response. In the current study, CTB adjuvant was added to the N-terminal of the vaccine peptide by EAAAK linker. CTB is the non-toxic component of the cholera toxin, which aids in the formation of a pentameric structure that binds to GM1-ganglioside receptors of the gut mucosa and controls the expression of receptors in gut tissue cells^39,60^. This toxin serves as a strong adjuvant when combined to other proteins and significantly elevates the ability of proteins to induce immune response after oral administration. Toxin derived adjuvant (such as CTB) has been used in several studies, where it was noted effective in enhancing the immunogenicity of multiepitope vaccines by inducing the production of immunoglobulins (IgG and IgA)^17,27,59,61,62^. The inclusion of non-toxic CTB to the designed vaccine enhances the efficacy of the potential peptides attached to it and promotes nasal or oral administration, and since the intestinal tissue damage observed in chickens infected by *Eimeria* results in severe dehydration and bloody diarrhoea, the combination of CTB and the multiepitope vaccine may counteract the damage caused by the parasite and reduce water loss by binding to epithelial receptors^63^.

The constructed vaccine was found to be immunogenic with a score of 8.59968, antigenic with VaxiJen score of 0.6043 and non-allergenic, making it a good candidate for vaccine development. The vaccine consists of 731 amino acid residues with a molecular weight of 73.25 kDa with an instability index of 33.40, fitting the criteria that vaccine proteins with molecular weight < 110 kDa and instability index < 40 are relatively stable/good vaccine candidates^22,27^. The evaluation of vaccine physiochemical properties further revealed the designed vaccine construct to be thermostable, acidic with a highly hydrophobic nature (GRAVY = 0.140); physiochemically fitting for production. Similar findings were observed by Ojha et al.^54^. The designed vaccine was subjected to structural validation using a Ramachandran plot and ProSA with scores of 83.2% and − 10.48, respectively. These scores indicated that the vaccine structure was of good quality, as the model lied in the vicinity of X-ray resolved structures in PDB.

Toll-like receptors (TLRs) are highly conserved transmembrane proteins that play a vital role in detecting and reducing foreign molecule within host such as pathogens^15^. These receptors detect protozoan parasites through recognition and activation of conserved pathogen components such as pathogen-associated molecular patterns (PAMPs)^45^. Activation of PAMPS within host signals TLR to produce and regulate the expression of cytokines, i.e. interleukin (IL)-12, interferons (IFNs), tumour necrosis factor (TNF), etc^45^. Production of these cytokines initiates the host's innate immune system to develop an adaptive immune response crucial from host protection against infection^62^. In the present study, the designed vaccine was docked to the TLR5 receptor. This receptor has been previously reported as an effective inducer of innate immune response in chickens infected with *Eimeria*^64^. With the majority of apicomplexans using the gliding motility when invading the host, TLR5 in *Eimeria* infections is likely to be induced by the presence of flagellin in the intestinal microbial flora and can easily be detected in chickens with mature intestinal gut^13,59,65^. The selection of the TLR5 as a receptor for the designed vaccine was informed by the fact that the receptor is both present in humans and chickens, where it responds to bacterial flagellin^66^. Though it is known that TLR5 responds to flagellin-like ligands, it has still been shown from gene expression profiling studies that TLR5 signalling pathway activation was detected after stimulation by cholera toxin B^40^. Moreover, it has also been confirmed that aside from flagellin, TLR5 also responds to profilin protein from the apicomplexan parasite-*Toxoplasma gondii*^67^. Interestingly, this profilin protein has also been reported to be expressed in all the developmental stages of several *Eimeria* parasite species and is conserved^68,69^.

Molecular docking of the vaccine candidate allowed analysis of binding interaction of vaccine to the receptor. The highest binding energy observed from the docking simulation revealed that the vaccine complex has a significant affinity to the receptor and can stimulate TLRs within the host leading to an improved immune response against *Eimeria*. Similar findings were obtained by Yin et al.^15^ when they evaluated *E. tenella* IMP1 and flagellin (TLR5 agonist) as potential *Eimeria* vaccine candidate. They proved through vaccination of three-week-old AA broiler chickens that recombinant EtIMP1-flagellin fusion protein enhanced the immune response of chickens, making it an effective immunogen^15^. Molecular docking results were further validated using molecular dynamics simulation, where obtained RMSD, RMSF and energy decomposition analysis revealed significant interaction of the vaccine construct and the TLR5^51,54^.

To assess the ability of the proposed vaccine to initiate immune response, the vaccine was subjected to an immune simulation using C-IMMSIM server. The *in silico* immune response results showed a consistent behaviour correlating with expected outcome from the host’s immune system when induced by vaccination. The production of IgA upon initial administration of vaccine into the simulation stimulated an increase in all immunoglobulin response where elevated production of antibodies that aid in secondary and tertiary response (i.e., IgG1 + IgG2, IgG1 and IgG2) was observed, followed by a significant reduction in antigen (Ag) concentration (Fig. 6a). The administration of the vaccine was also noted to elevate the B-cell population, especially the memory B-cell (Fig. 6b). The gradual increase of B-cell memory cells and antibodies after subsequent exposure to the vaccine injections confirmed the vaccine's effectiveness to host when exposed for a duration of time, consistent with the vaccine’s immunogenicity. Similar behaviour was observed in the T cytotoxic(TC) cells and T helper (TH) response, where memory cells were elevated (Fig. 6c,d). The CD8^+^ cytotoxic cells and CD4^+^ T helper cells are critical for anticoccidial immunity against avian coccidiosis, and it has been reported that increased populations of T cells are linked to increased production of pro-inflammatory cytokine interferon (IFN)-γ, capable of regulating and inhibiting the development of *Eimeria* parasite^70^.

IFN-γ is an important cytokine in chickens known to enhance expression of MHC II antigen and is involved in Th1-mediated immune response^71^. The immune simulation showed elevated production of IFN-γ, TGF-β and IL-2 (see Supplementary Fig. S1a). The increased, continuous production of cytokines such as interferon (IFN)-γ and T-cells show the vaccine’s potential to exert a protective effect against parasite infections since they are crucial for cellular immune response and anticoccidial immunity towards coccidiosis^19,72^. Zhang et al.^73^ reported upregulation of TL5 in *E. tenella* infection, which triggered activation of pro-inflammatory cytokines IL-2 and IFN-γ. This supports immune simulation findings obtained in this study, where vaccine construct induced increased production of IFN- γ and IL genes (see Supplementary Fig. S1a). Similar findings were reported by Liu et al.^74^ in a separate study, evaluating the efficacy of DNA vaccine (pVAX-Ea14–3–3) against *Eimeria* infection. The authors confirmed production of IFN- γ, IL-2 and IL-4 in high levels provided effective protection against *Eimeria* infection (*E. acervulina, E. maxima and E. tenella*). The elevated levels of IFN- γ enhances vaccine efficacy through induction of microbicidal effects, crucial in resolving parasitic infections. High levels of cytokines in the host activate microphages that inhibit and kill *Eimeria* while enhancing immune responses. As observed in this study, subsequent doses of vaccine activated the growth of macrophages (see Supplementary Fig. S1b) and cytotoxic T cells (Fig. 6c), which remained elevated throughout the simulation.

Avian coccidiosis immunity is linked to innate and adaptive, where the innate immune response to *Eimeria* parasites is activated at different phases of the parasite life cycle and is facilitated by natural killer (NK) cells, dendritic cells, epithelial cells, heterophils, and macrophages^70^. Innate immune response against *Eimeria* infection is activated at the early stages of the infection and serves as the first line of defence, where it utilises immune receptors to detect and respond to parasitic infection^45^. The adaptive immune response is crucial for the prevention of host invasion and growth of the parasites. This mode of immunity in chickens is specific, encompassing both cellular and humoral immune mechanisms and is regulated by B- and T-cells. T-cells have been reported to produce cytokines such as interleukin (IL)-2, IL-4, IL-10, IL-12 and IL-18, tumour necrosis factor (TNF)-α and transforming growth factor (TGF)-β1–4, as a part of protective immunity against avian coccidiosis^75^. These findings correlate with our immune system simulation results (Fig. S1a), confirming the potential of the proposed multiepitope vaccine in inducing long-lasting humoral and cellular immune responses and protection against *Eimeria* infections. Previous literature suggests that *in silico* immune simulations can be consistent with the real immune response exhibited by the affected host against pathogens^56^. The simulation outcome for the present study is a crucial step in vaccine development and could potentially provide reliable insight on the efficiency of the designed vaccine against *Eimeria* through induction of protective immune response. Also, the optimized expression of the proposed vaccine in the *E. coli* K12 strain and successful *in silico* cloning into expression vector promises for easier and more accurate production of vaccine in large scales^76^. However, findings obtained in this study still require further laboratory experiments for validation purposes.

## Conclusion

From the present study, the design of a multiepitope vaccine was achieved successfully using the immunoinformatic approach. The vaccine designed exhibited all the parameters crucial for potential vaccine candidates, as it effectively induced immune response through the production of cytokines in an immune simulation technique. Based on this study, it might be promising to focus on specific regions of the parasite’s protein rather than large protein residues, as this might contribute to the reduction of the parasite’s antigen complexity. Also, the combination of multiple T-cells from different phases of the *Eimeria* life cycle may effectively confer ideal protection against multiple *Eimeria* species though this still requires further experimental validation(s). This would also minimise any possible negative effects of using the whole genome of the parasite, reducing risk of reinfection. In the present study, a multiepitope vaccine candidate containing 7 CTL epitopes, 21 HTL epitopes and 6 B-cell epitopes, and an adjuvant resulted in a vaccine construct that significantly enhanced immune protection of the host by prediction. It can be concluded that the immunoinformatic approaches explored in the prediction of the designed vaccine candidate yielded promising results. It is highly recommended that further studies and experimental validation be done on the results obtained and reported in the current study for confirmation purposes and to validate the safety and efficacy of the vaccine.

## Supplementary Information

Supplementary Information.
