# Author Correction: Designing multiepitope-based vaccine against *Eimeria* from immune mapped protein 1 (IMP-1) antigen using immunoinformatic approach

**DOI:** 10.1038/s41598-022-20081-2

**Published:** 2022-09-13

**Authors:** Thabile Madlala, Victoria T. Adeleke, Abiodun J. Fatoba, Moses Okpeku, Adebayo A. Adeniyi, Matthew A. Adeleke

**Affiliations:** 1 Discipline of Genetics, School of Life Sciences, University of KwaZulu-Natal, P/Bag X54001, Westville, Durban, 4000 South Africa; 2 Discipline of Chemical Engineering, University of KwaZulu-Natal, Howard Campus, Durban, 4041 South Africa; 3 Department of Chemistry, Faculty of Natural and Agricultural Sciences, University of the Free State, Bloemfontein, South Africa; 4 Department of Industrial Chemistry, Federal University Oye-Ekiti, Oye-Ekiti, Nigeria

Correction to: *Scientific Reports* 10.1038/s41598-021-97880-6, published online 14 September 2021

The original version of this Article contained an error in the Results section under the subheading ‘Prediction of cytotoxic T-cell/CD8^+^ T-cell epitopes’, where an incorrect Vaxijen score was stated for the epitope ‘FKISVFGFA’.

“A total of 77 CTL epitopes were detected to be antigenic in nature, with epitope ‘FKISVFGFA’ having the highest Vaxijen score of 2 0.2931.”

now reads:

“A total of 77 CTL epitopes were detected to be antigenic in nature, with epitope ‘FKISVFGFA’ having the highest Vaxijen score of 2.2931.”

The original Article has been corrected.

