# Supplementary material for: Designing multiepitope-based vaccine against Eimeria from immune mapped protein 1 (IMP-1) antigen using immunoinformatic approach
Source: Sci Rep. 2021 Sep 14;11:18295. doi: 10.1038/s41598-021-97880-6 (PMC8440781; doi:10.1038/s41598-021-97880-6)
Supplement: Supplementary file 1 — Supplementary Information. [file 41598_2021_97880_MOESM1_ESM.pdf]

Designing multiepitope-based vaccine against *Eimeria* from Immune mapped protein 1 (IMP-1) antigen using immunoinformatic approach

Table S1: Eimeria IMP1 protein sequences and their species

Sequence ID Name	Name of Species
>AGG13402.1	immune mapped protein 1 [*Eimeria tenella*]
>XP_013228705.1	Immune mapped protein 1 [*Eimeria tenella*]
>CDJ37867.1	Immune mapped protein 1 [*Eimeria tenella*]
>CBL80638.2	immune mapped protein 1 [*Eimeria tenella*]
>CBL80642.1	immune mapped protein 1 [*Eimeria maxima*]
>CBL80637.1	immune mapped protein 1 [*Eimeria maxima*]
>CBL80636.1	immune mapped protein 1 [*Eimeria maxima*]
>AKJ88134.1	immune-mapped protein 1 [*Eimeria maxima*]
>XP_013350261.1	Immune mapped protein-1, related [*Eimeria mitis*]
>XP_013334350.1	Immune mapped protein-1, related [*Eimeria maxima*]
>XP_013250345.1	Immune mapped protein-1, related [*Eimeria acervulina*]
>CDJ48735.1	Immune mapped protein-1, related [*Eimeria brunetti*]
>CDJ57702.1	Immune mapped protein-1, related [*Eimeria maxima*]
>CDI79570.1	Immune mapped protein-1, related [*Eimeria acervulina*]
>CDJ27683.1	Immune mapped protein-1, related [*Eimeria mitis*]
>XP_013435884.1	Immune mapped protein 1, related [*Eimeria necatrix*]
>CDJ67417.1	Immune mapped protein 1, related [*Eimeria necatrix*]
>CDI75999.1	Immune mapped protein 1, related [*Eimeria praecox*]

(a)

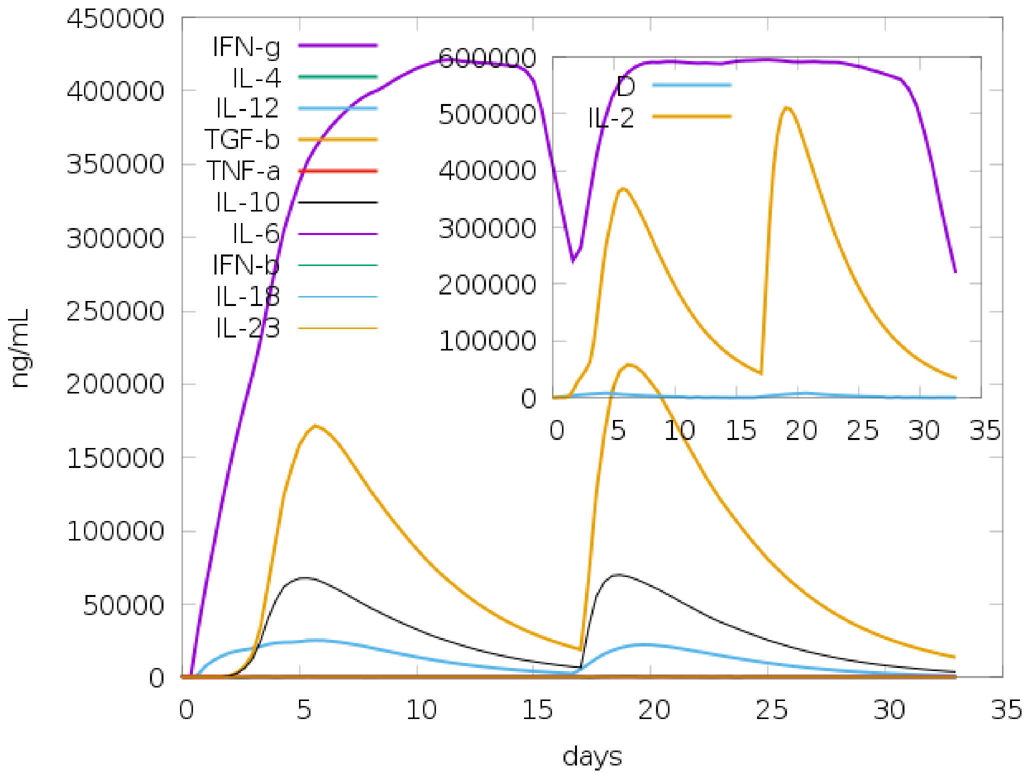

(b)

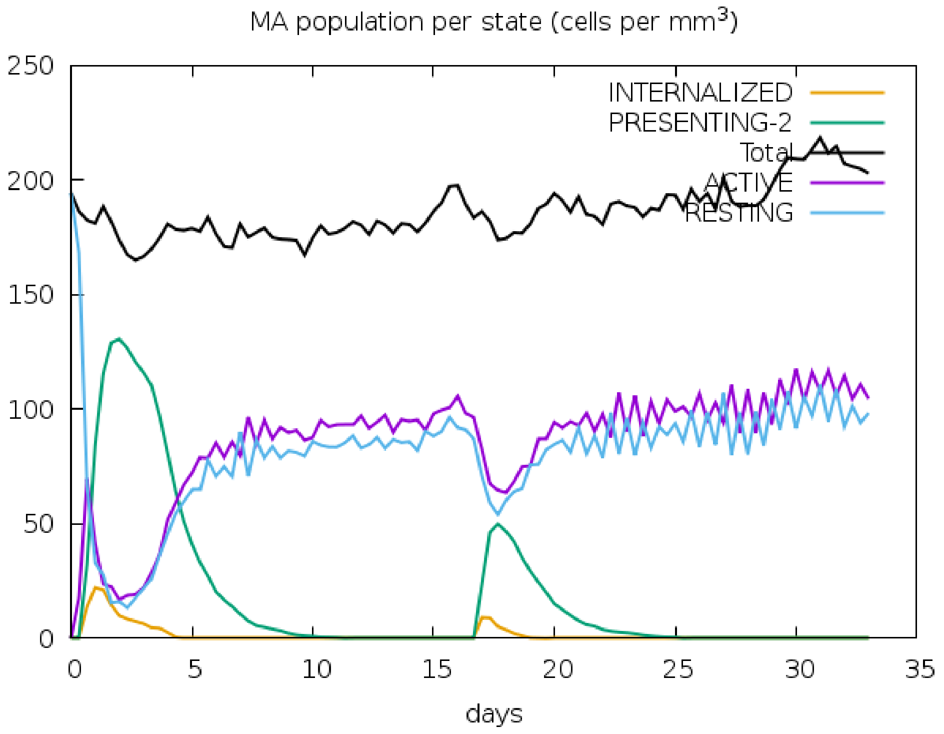

Fig. S1 (a) Cytokine plot showing Concentration of cytokines and interleukins. D in the inset plot is danger signal. **(b)** Macrophage plot showing total count, internalized, presenting on MHC class-II, active and resting macrophages.
